# Astragaloside IV supplementation attenuates cognitive impairment by inhibiting neuroinflammation and oxidative stress in type 2 diabetic mice

**DOI:** 10.3389/fnagi.2022.1004557

**Published:** 2022-09-29

**Authors:** Yaxuan Zhang, Yuan Yuan, Jiawei Zhang, Yao Zhao, Yueqi Zhang, Jianliang Fu

**Affiliations:** Department of Neurology, Shanghai Jiao Tong University Affiliated Sixth People’s Hospital, Shanghai, China

**Keywords:** astragalosides IV, cognitive impairment, type 2 diabetes mellitus, neuroinflammation, oxidative stress

## Abstract

Although diabetic cognitive impairment is one of the most common complications of type 2 diabetes mellitus (T2DM), optimized therapeutic strategies are not available yet. Astragalosides IV (AS-IV) is a traditional Chinese medicine possessing diverse pharmacological properties including anti-inflammatory and antioxidant effects. However, the effects of AS-IV on diabetes-related cognitive impairment and its precise mechanisms remain largely unknown. T2DM mice, induced by a high-fat diet (HFD) and an intraperitoneal injection of low-dose streptozotocin (STZ) were administrated with AS-IV every other day for eight consecutive weeks. Learning and memory abilities were assessed subsequently using the Ymaze test and the anxious behavior was evaluated using an open field test. Then, the morphology and number of neurons and microglia were observed by HE staining or immunohistochemistry. Oxidative stress biomarkers and pro-inflammatory cytokines were determined using relevant kits. In addition, the expression levels of Nrf2, Keap1, HO-1, and NQO1 were determined by Western blot analyses. The results indicated that AS-IV administration significantly improved neuronal damage and cognitive deficit in T2DM mice. Meanwhile, oxidative stress and neuroinflammation were also ameliorated in T2DM mice, which might be attributed to the regulation of Nrf2/Keap1/HO-1/NQO1 pathway in T2DM mice. Taken together, these data suggested that AS-IV ameliorates cognitive impairment in T2DM mice by attenuating oxidative stress and neuroinflammation, possibly through modulating the Nrf2/Keap1/HO1/NQO1 pathway.

## Introduction

Type 2 diabetes mellitus (T2DM) is a lifelong metabolic disease that results in various complications and is thus becoming a main public health problem. Diabetes-related cognitive impairment is a common but grossly underestimated complication of T2DM (Biessels and Despa, 2018; Srikanth et al., 2020). T2DM has been reported to significantly accelerate the deterioration of cognitive function. Clinical and epidemiological studies have also demonstrated that patients with T2DM have nearly twice the risk of developing dementia (Feil et al., 2011). Given the prevalence of T2DM, the incidence of diabetes-related cognitive impairment is expected to surge and cause an unpredictable social and economic burden. Thus, it is of great importance to decipher the underlying mechanisms of diabetes-related cognitive impairment and to develop effective therapeutic agents for the disease.

An increasing number of studies have indicated that oxidative stress and neuroinflammation are key contributors to the initiation and progression of diabetes-related cognitive impairment (Piatkowska-Chmiel et al., 2021; Wang et al., 2021; Wu et al., 2022). For example, increased oxidative stress was associated with neuronal damage and memory deficit in T2DM (Hoyos et al., 2022). Besides, excessive neuroinflammation leading to a cognitive deficit was also observed in T2DM (Marioni et al., 2010; Zhao et al., 2019; Ko et al., 2021). Nuclear factor erythroid 2-related factor 2 (Nrf2) is a redox-sensitive transcription factor that has been proved to be a master regulator of both neuroinflammation and oxidative stress (Ma, 2013; Ahmed et al., 2017). A growing body of studies has established the protective role of Nrf2 against oxidative stress and neuroinflammation in T2DM. It has been reported that Nrf2 increases the transcription of heme oxygenase-1 (HO-1) and NAD(P)H: quinone oxidoreductase 1 (NQO1) that mitigates the oxidative stress and inflammation of the brain and eventually improves the cognitive impairment in T2DM (Feng et al., 2018; Wang G. et al., 2020; Pang et al., 2021).

Astragaloside IV (AS-IV), a major active component extracted from Astragalus membranaceus, exerts multiple pharmacological activities including anti-inflammation and anti-oxidation (Gui et al., 2013b; Zhang and Frei, 2015). The protective effect of AS-IV against diabetes complications and neurological disease has been widely investigated in numerous literatures (Qiao et al., 2017; Wang et al., 2017, Wang E. et al., 2020; Xia et al., 2020). However, whether AS-IV treatment improves diabetes-related cognitive impairment and if so, what are the underlying mechanisms, are still unrevealed. Therefore, in the present study, we explored the hypothesis that AS-IV might improve diabetes-related cognitive impairment in T2DM mice by modulating the Nrf2/Keap1/HO-1/NQO1 pathway.

## Materials and Methods

### Animals and experimental protocols

Four-week-old male C57BL/6J mice were housed in an SPF-barrier environment under standard conditions of temperature (22 ± 2°C), humidity (55%–65%), and light (12:12 h light/dark cycle) and were randomly divided into three groups: wild type group (WT; *n* = 10), T2DM model group (T2DM; *n* = 10) and T2DM + AS-IV treatment group (AS-IV; *n* = 10). The mice in the WT group were given a normal diet (10% calories from fat; Research Diet, New Jersey, United States), while the mice in the other groups were fed with HFD (60% calories from fat; Research Diet, New Jersey, United States) during the whole experimental period. After 4 weeks of HFD (Zhu et al., 2022), mice in the T2DM and AS-IV group were fasted for 8 h overnight and then injected intraperitoneally with STZ (35 mg/kg, dissolved at 0.1 mm cold citrate buffer, pH 4.4; Yeasen Biotechnology, Shanghai, China) for three consecutive days to induce T2DM (Zhang et al., 2018). The mice were considered diabetic when the blood glucose levels were higher than 11.6 mmol/L 3 days after STZ injection. Then, referring to previous reports, mice in AS-IV group were intragastrically administered with 40 mg/kg of AS-IV (Song et al., 2018; Yuanye Biotechnology, Shanghai, China) every other day for 8 weeks while mice in T2DM and WT group were intragastrically administered with a vehicle. The fasting blood glucose levels of each mouse in all groups were tested every 4 weeks. All animal experiments were carried out according to the ethical committee on animal welfare of Shanghai Jiao Tong University Affiliated Sixth People’s Hospital and the principles outlined in the National Institutes of Health (NIH) Guide for the Care and Use of Laboratory Animals.

### Y maze test

Learning and memory abilities were measured using a Y maze apparatus that was made up of three similar arms (A, B, C) with dimensions of 33 cm long, 15 cm high, and 10 cm wide at 120° angles to each other. After at least 30 min of acclimatization in the test room, the mouse was placed in the center of the apparatus and was allowed to move freely for 5 min. Spontaneous alternation was evaluated by the pattern of complete entry into each arm (the mouse’s hind paws go entirely into the arm). Actual alternation was defined as the number of consecutive entries into the three arms on overlapping triplet sets, and the alternation behavior (%) was calculated as follows (You et al., 2020):


Alternation behavior (%) = (Actual alternation)/(Total number of arms entries – 2)

### Open field test

Anxiety-like behavior was measured using an open field apparatus of 50 cm long, 50 cm wide, and 50 cm high. After at least 30 min of acclimatization in the test room. Each mouse was placed in the center of the apparatus and was allowed to explore freely for 5 min. The apparatus was disinfected with 75% alcohol after each test. The central area was defined as half of the total area located in the center of the apparatus. Time spent in the center area and the number of times entering the center area of each mouse were recorded for data analysis (Yoshizaki et al., 2020).

### Hematoxylin and eosin staining

After the behavioral tests of cognitive ability, mice were anesthetized deeply and transcardially perfused with 400 ml phosphate buffer saline (PBS) followed by 400 ml 4% paraformaldehyde. Then, the brain was removed and postfixed in 4% paraformaldehyde for 24 h at 4°C. After dehydration in graded series of alcohol, the brain tissue was embedded in paraffin and cut into 5 μm thickness. The sections were further stained with hematoxylin and eosin and were observed under a light microscope (IX53, Olympus, Tokyo, Japan).

### Dihydroethidium staining

After postfixed for 24 h, the formalin preserved brain tissue was kept in 30% sucrose until it sank to the bottom. Then, the brain tissue was embedded in OCT and cut into 10 μm thick frozen sections. Dihydroethidium (DHE) staining detecting ROS level in situs was performed as previously described (Zhao et al., 2021). Briefly, the brain sections were incubated with 10 mmol/L DHE (Sigma-Aldrich, St. Louis, MO, USA) at 37°C for 30 min in a humidified chamber protected from light. 1 μg/ml 4,6-diamidino-2-phenylindole, dilactate (DAPI; Thermo Scientific, Waltham, MA, USA) was used before the sections were coverslipped. The brain sections were then visualized under a fluorescence microscope (IX53, Olympus, Tokyo, Japan).

### MDA level and SOD activity evaluation

MDA is one of the end products of lipid peroxidation and is considered as a marker of oxidative stress, while SOD activity is used as an antioxidant marker. Hippocampal tissues of different groups were homogenized to obtain the supernatant for subsequent analyses and the level of MDA and SOD activity was detected using biochemical assay kits (Nanjingjiancheng, Nanjing, China) following the manufacturer’s protocol.

### Immunohistochemistry

To detect the status of microglia, immunohistochemical staining was performed. After deparaffinization and antigen retrieval (0.05% citraconic acid), the sections were treated with endogenous peroxidase (3% H_2_O_2_ in PBS) for 10 min, followed by a blocking buffer containing 10% bovine serum albumin in PBS for 1 h. Next, the sections were incubated with the antibody of ionized calcium-binding adapter molecule 1 (Iba1; Abcam, Cambridge, United Kingdom) overnight, and followed by incubation with biotinylated secondary antibodies (1:500) for 30 min the following day. The sections were then incubated with the avidin-biotin complex (Vector Laboratories, Burlingame, CA, USA) for 30 min, and visualized by 3,3-diaminobenzidine (DAB; Vector Laboratories) reaction. After dehydration in ethanol and clarification in xylene, the sections were visualized under a microscope (IX53, Olympus, Tokyo, Japan).

### ELISA assays

The levels of pro-inflammatory cytokines including tumor necrosis factor alpha (TNF-α), interleukin 6 (IL-6), and interleukin one beta (IL-1β) of cerebral hemispheres were determined using ELISA kits (Multisciences, Hangzhou, China) following the procedures of the manufacturer.

### Western blot analysis

Samples of hippocampal tissue of different groups stored at −80°C were homogenized in prechilled radioimmunoprecipitation assay (RIPA) buffer containing protease and phosphatase inhibitors (Beyotime, Shanghai, China). The protein concentration of the samples was determined using the BCA kit (Beyotime, Shanghai, China). The same amounts (20–30 μg) of brain protein were separated by sodium dodecyl sulfate-polyacrylamide gel electrophoresis (SDS-PAGE) on 7.5% or 12% gels and then transferred onto a polyvinylidene difluoride membranes (PVDF). The membranes were incubated overnight at 4°C with different primary antibodies. The primary antibodies used were as follows: Nrf2 (1:1,000, Abcam), Keap1(1:1,000, Abcam), HO-1(1:1,000, ABclonal), NQO1(1:1,000, Servicebio), and β-actin (1:5,000, CST). The next day, the membranes were incubated with relevant secondary antibodies at room temperature for 1 h. The immunocomplexes were detected using a chemiluminescence reagent (Thermo Scientific, Waltham, MA, USA) with automatic chemiluminescence apparatus (BIO-RAD, USA) and the images with a specific molecular band were analyzed using ImageJ.

### Statistical analysis

Data in our study were expressed as the mean ± standard error of the mean (SEM). Statistical significance was assessed by two-way ANOVA with posttest (Turkey) for data of fasting blood glucose, and the other data were assessed by one-way ANOVA with posttest (Turkey). Values of *P* < 0.05 were considered statistically significant. Statistical data were analyzed using GraphPad Prism 7.0.

## Results

### Astragalosides IV ameliorates the hyperglycemia in T2DM mice

**Figure 1:**
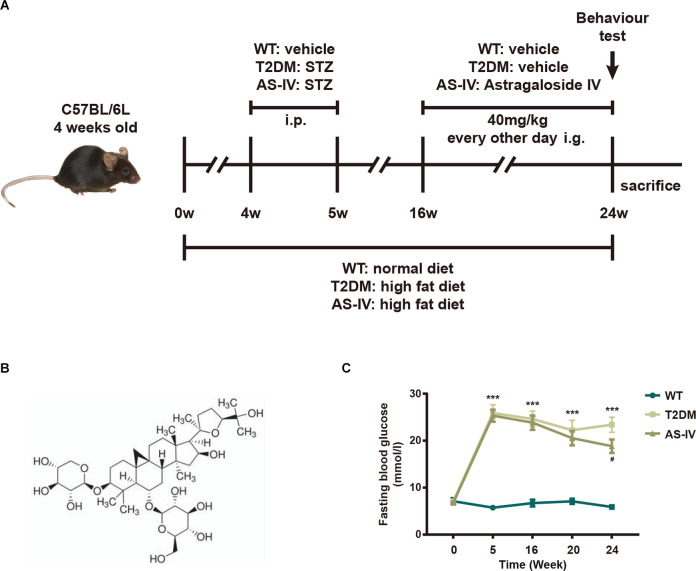
Effects of AS-IV on body weight and fasting blood glucose of type 2 diabetes mellitus (T2DM) mice. **(A)** The experimental design scheme for high-fat diet (HFD)/STZ-induced T2DM. Male 4-week-old C57BL/6J mice were fed with either the normal diet or HFD for 4 weeks. The HFD-fed mice were injected intraperitoneally (i.p.). with freshly prepared STZ (35 mg/kg) for 3 days to induce T2DM. From week 16 the T2DM mice were intragastrically (i.g.) administered with AS-IV (40 mg/kg) or vehicle for another 8 weeks. WT mice on a normal diet received vehicle administration. **(B)** 2-D structure of AS-IV. **(C)** Fasting blood glucose levels. Data are presented as the mean ± standard error of the mean (SEM; *n* = 8). **P < 0.01; ***P < 0.001 T2DM vs. WT; #*P* < 0.05; ###*P* < 0.001 AS-IV vs. T2DM.

The experimental design is shown in Figure 1A. After 4 weeks of HFD treatment, mice in T2DM and AS-IV groups were injected with STZ for 3 days. One week after the STZ injection, the fasting blood glucose levels of the mice in T2DM and AS-IV groups were detected. The results showed that fasting blood glucose levels of the mice were higher than 11.6 mmol/L, which suggests that the T2DM model was successfully established (Figure 1B). To evaluate whether AS-IV had effects on the metabolic parameters of T2DM mice, the fasting blood glucose levels of mice in each group were detected. We found that the fasting blood glucose levels were consistently high in T2DM mice, while decreased fasting blood glucose levels were observed after 8 weeks of AS-IV treatment (Figure 1C). Therefore, these findings illustrated that AS-IV treatment could partly reverse the glucose metabolism abnormalities in T2DM mice.

### Astragalosides IV improves cognitive impairment in T2DM mice

**Figure 2:**
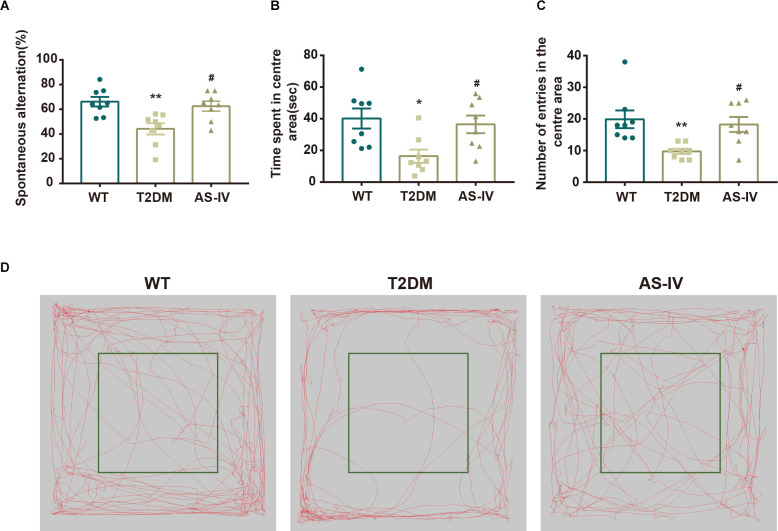
AS-IV ameliorated cognitive impairment in T2DM. **(A)** The representative exploring traces of mice in the open field. **(B)** The percentage of spontaneous alternation in the Y maze. **(C)** Time spent in the center area of the open field. **(D)** Number of entries in the center area of the open field. Data are presented as the mean ± SEM (*n* = 8). *P < 0.05; **P < 0.01 T2DM vs. WT; #*P* < 0.05 AS-IV vs. T2DM.

Y maze test and Open field test were performed to evaluate the effects of AS-IV on cognitive impairment in T2DM mice. Y maze test, based on the general tendency of rodents to explore new environments, was used to evaluate the learning and memory abilities of mice in each group. Mice with normal learning and memory abilities usually tend to alternate among the three arms of the Y maze (You et al., 2020). However, the results showed that compared to the WT group, the percentage of spontaneous alternations in the T2DM group was significantly decreased, while AS-IV treatment reversed the decline (Figure 2A). Meanwhile, an Open field test was used to investigate the autonomous exploratory behavior of mice in the new environment, which can reflect the anxiety phenotype of mice. An increase in the frequency of exploration behavior often means a decrease in anxiety phenotype. Compared to the WT group, mice in T2DM group tended to spend less time in the center area and the number of entries in the center area was also decreased, while AS-IV treatment significantly reversed the decrease (Figures 2B–D). Together, these results indicated that AS-IV treatment improved cognitive impairment including learning and memory deficiency and anxiety in T2DM mice.

### Astragalosides IV attenuates neuronal damage in T2DM mice

**Figure 3:**
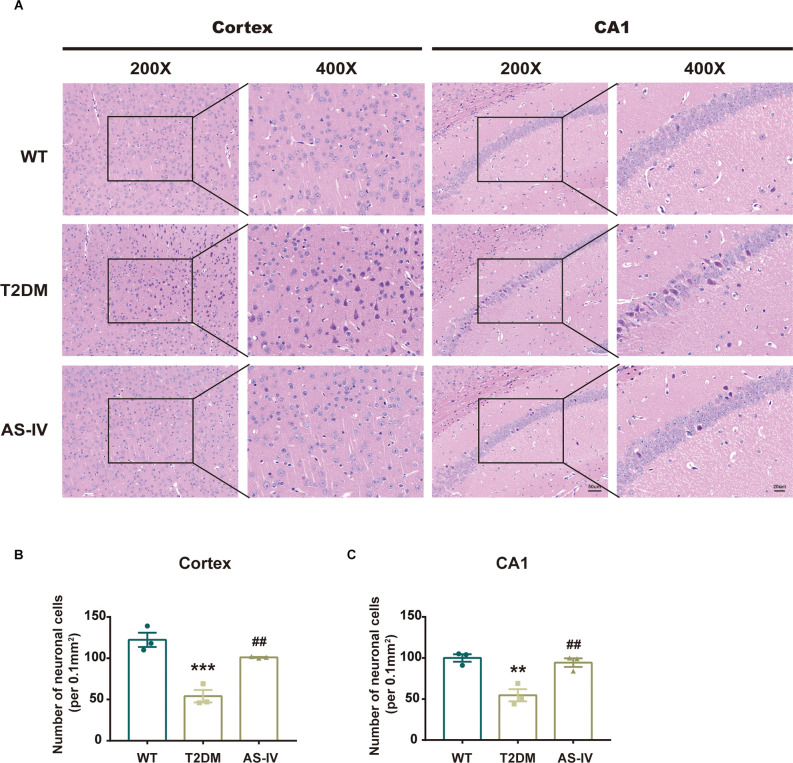
AS-IV reduced neuronal death in the cortex and hippocampus CA1 of T2DM mice. **(A)** Represented photomicrographs of HE staining in the cortex and hippocampus CA1 (magnification ×200 or ×400, scale bar = 50 μm or 20 μm). **(B)** Number of neuronal cells in the cortex. **(C)** Number of neuronal cells in hippocampus CA1. Data are presented as the mean ± SEM (*n* = 3). **P < 0.01; ***P < 0.001 T2DM vs. WT; ##*P* < 0.01 AS-IV vs. T2DM.

HE staining was used to detect the change in the number and morphology of neurons in the cortex and hippocampal CA1 regions that play important roles in memory formation and retrieval (Zhao et al., 2021). We found that AS-IV treatment noticeably reversed the reduction in neuronal number in T2DM mice (Figures 3A–C). Compared with the WT group, neurons of mice in T2DM group were shrunk and the nucleus was deeply stained, while AS-IV treatment rescued these morphological abnormalities in neurons of T2DM mice (Figure 3A). These findings indicated that AS-IV treatment alleviated neuronal damage in T2DM mice.

### Astragalosides IV alleviates oxidative stress in T2DM mice

**Figure 4:**
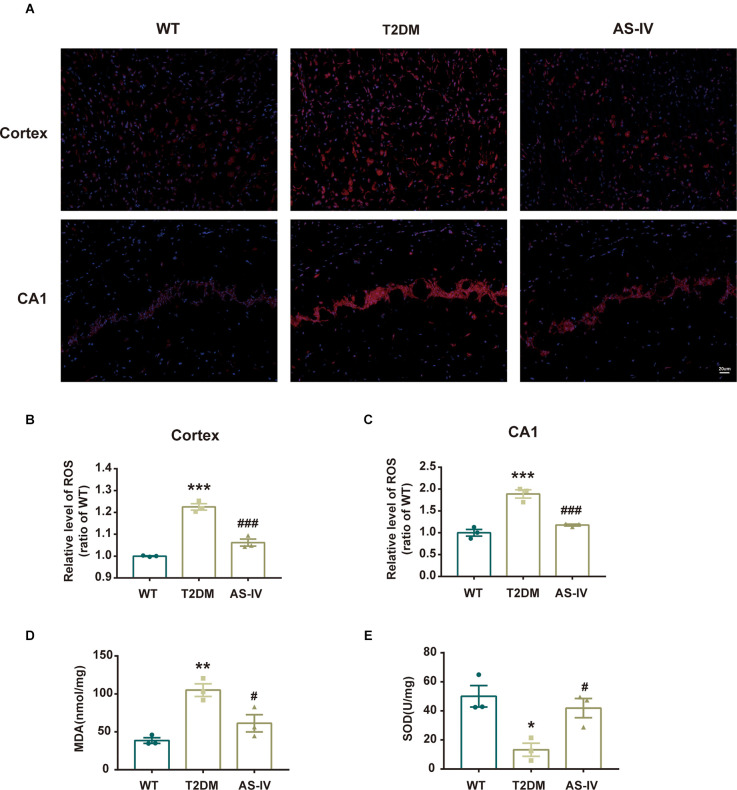
AS-IV ameliorated oxidative stress in T2DM mice. **(A)** Represented photomicrographs of dihydroethidium (DHE) staining for ROS in the cortex and hippocampus CA1 (magnification ×200, scale bar = 50 μm). **(B)** The relative level of ROS (ratio to WT group) in the cortex. **(C)** The relative level of ROS (ratio to WT group) in hippocampus CA1. **(D)** MDA levels of the hippocampus . **(E)** SOD activity of the hippocampus. Data are presented as the mean ± SEM (*n* = 3). *P < 0.05; **P < 0.01; ***P < 0.001 T2DM vs. WT; #*P* < 0.05; ###*P* < 0.001 AS-IV vs. T2DM.

Oxidative stress is implicated in neuronal damage in various neurological diseases (Chong et al., 2005). To examine whether AS-IV could protect the brain tissue from oxidative stress damage in T2DM, the ROS, MDA levels, and SOD activities were measured. The ROS levels were detected using DHE staining and the results showed that AS-IV treatment markedly ameliorated ROS increase in the cortex and hippocampus CA1 of T2DM mice (Figures 4A–C). In addition, MDA levels and SOD activities of the hippocampus were also detected using the relevant kits. In T2DM group, MDA levels were dramatically increased, while SOD activities were dramatically decreased. Treatment with AS-IV reduced MDA levels and increased SOD activities of T2DM mice (Figures 4D,E). Collectively, these data demonstrated that AS-IV treatment attenuated oxidative stress in T2DM mice.

### Astragalosides IV mitigates neuroinflammation in T2DM mice

**Figure 5:**
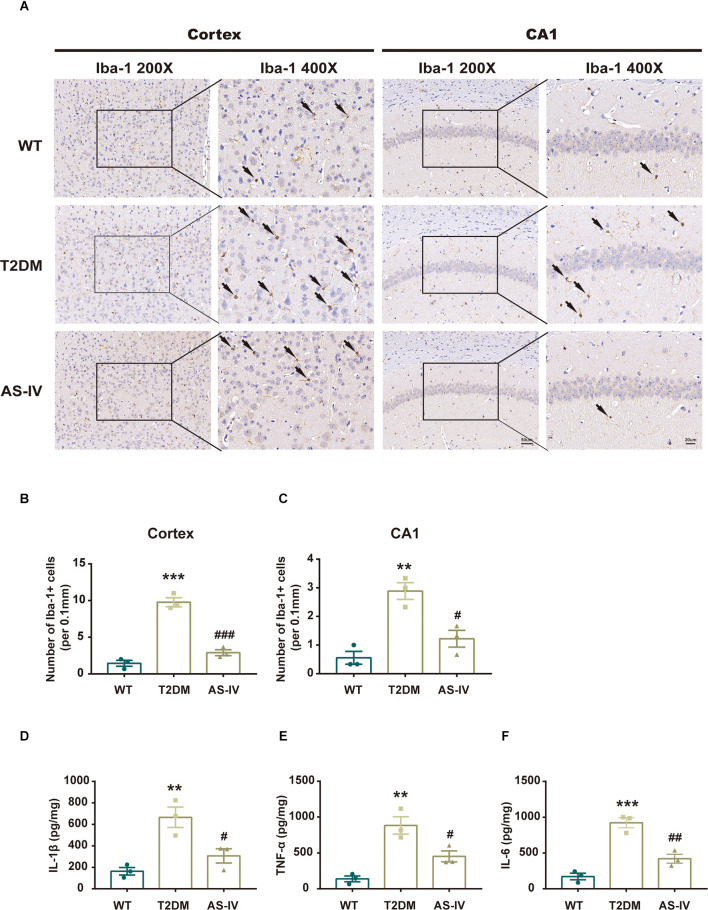
AS-IV reduced microglia activation and pro-inflammatory cytokines production in T2DM mice. **(A)** Represented photomicrographs of immunohistochemistry using Iba-1 in the cortex and hippocampus CA1 (magnification ×200 or ×400, scale bar = 50 μm or 20 μm). **(B)** Number of Iba-1 positive cells in the cortex. **(C)** Number of Iba-1 positive cells in hippocampus CA1. **(D)** IL-1β levels of the hippocampus. **(E)** TNF-α levels of the hippocampus. **(F)** IL-6 levels of the hippocampus. Data are presented as the mean ± SEM (*n* = 3). **P < 0.01; ***P < 0.001 T2DM vs. WT; #*P* < 0.05; ##*P* < 0.01; ###*P* < 0.001 AS-IV vs. T2DM.

Microglia are innate immune cells of the central nervous system that play an important role in the development of neuroinflammation. Therefore, to investigate whether T2DM could promote microglia activation and the subsequent production of neurotoxic pro-inflammatory cytokines, we analyzed the number and morphology of microglia in cortex and hippocampus CA1 using immunohistochemistry. We found that T2DM produced an increase of Iba-1-positive microglia that had been transitioned from a lacy, highly ramified morphology indicative of a quiescent state to morphology with shortened processes and larger size, indicative of inflammatory activation (Figures 5A–C). After 8 weeks of treatment with AS-IV, the number and morphological changes of microglia in T2DM mice were significantly improved (Figures 5A–C). Furthermore, we detected the level of pro-inflammatory cytokines including IL-1β, IL-6, and TNF-α of hippocampus using ELISA kits. We found that when compared with the WT group, the levels of IL-1β, IL-6, and TNF-α were much higher in T2DM group, while AS-IV treatment suppressed the levels of pro-inflammatory cytokines (IL-1β, IL-6, and TNF-α) in the brain (Figures 5D–F). Overall, these findings indicated that AS-IV exerts anti-inflammatory effects in T2DM mice.

### The effects of astragalosides IV were involved in Nrf2/Keap1/HO-1/NQO1 pathway

**Figure 6:**
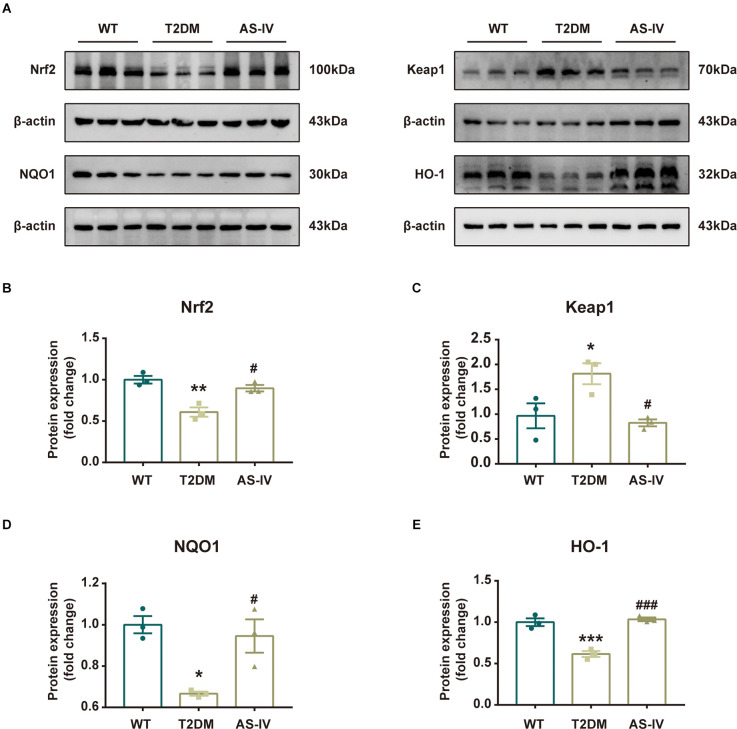
Nrf2/Keap1/HO-1/NQO1 signaling pathway was involved in the protective effect of AS-IV. **(A)** Representative bands of Western blot data. **(B)** Quantitative analysis of the Western blot bands of Nrf2. **(C)** Quantitative analysis of the Western blot bands of Keap1. **(D)** Quantitative analysis of the Western blot bands of NQO1. **(E)** Quantitative analysis of the Western blot bands of HO-1. Data are presented as the mean ± SEM (*n* = 3). *P < 0.05; **P < 0.01; ***P < 0.001 T2DM vs. WT; #*P* < 0.05; ###*P* < 0.001 AS-IV vs. T2DM.

To further reveal the mechanism underlying the neuroprotective effects of AS-IV on T2DM mice, we measured the expression levels of Nrf2 pathway-related proteins including Nrf2, Keap1, HO-1, and NQO1 using Western blot analysis. As the results showed, compared to the mice in the WT group, the protein expressions of Nrf2, HO-1, and NQO1 in T2DM mice were significantly decreased while the protein expression level of Keap1 was increased (Figures 6A–E). After 8 weeks of treatment of AS-IV, the protein expression levels of Nrf2, HO-1, and NQO1 were all increased, while the protein expression level of Keap1 was decreased (Figures 6A–E). These findings suggested that the neuroprotective effects of AS-IV on T2DM mice might be due to the Nrf2/Keap1/HO-1/NQO1 pathway.

## Discussion

Diabetes and cognitive impairment are two highly prevalent chronic disorders that frequently coexist in people older than 65 years old worldwide (Srikanth et al., 2020). Currently, increasing evidence shows that T2DM is an independent risk factor for cognitive impairment (Biessels et al., 2006; Biessels and Despa, 2018). However, the mechanisms by which T2DM causes cognitive impairment remain largely unknown, and there are still no effective strategies to prevent or delay the progression of cognitive impairment in T2DM (Srikanth et al., 2020). Thus, it is of great importance to find effective drugs for the treatment of diabetes-related cognitive impairment. AS-IV is a multifunction molecule with great oral bioavailability and brain-blood barrier penetration (Wang E. et al., 2020). The protective effects of AS-IV have been extensively studied in various neurodegenerative diseases and diabetic complications. It has been reported that AS-IV improves cognitive dysfunction in Alzheimer’s disease (AD) and chronic cerebral hypoperfusion-induced dementia (Kim et al., 2015; Wang et al., 2017). Moreover, a previous study reported that AS-IV improved blood glucose levels and attenuated renal dysfunction in diabetic rats (Zhang Y. et al., 2020). However, its role in diabetes-related cognitive impairment remains unknown. Therefore, in this study, we sought to explore whether AS-IV exerted neuroprotective effects on T2DM-related cognitive impairment and the possible mechanisms involved. Herein, a combination of HFD and low-dose STZ, which closely resembles human T2DM development, was used to induce T2DM in mice (Chen et al., 2022). We observed that AS-IV treatment slightly reduced the blood glucose levels and significantly improved cognitive dysfunction in T2DM mice.

Numerous studies have reported that both oxidative stress and neuroinflammation are the underlying pathogeneses of diabetes-related cognitive impairment (Piatkowska-Chmiel et al., 2021; Hoyos et al., 2022; Wu et al., 2022). Oxidative stress is one of the major factors that contribute to the development of cognitive impairment in T2DM. Multiple studies have demonstrated that excessive oxidative stress accompanied by impaired antioxidant capacity in T2DM encouraged the generation of ROS that further led to neuronal damage and cognitive dysfunction in T2DM (Valko et al., 2007; Gocmez et al., 2019; Michailidis et al., 2022). Furthermore, studies have shown that AS-IV exerts neuroprotective effects on AD and chronic cerebral hypoperfusion-induced dementia depending on the inhibition of oxidation stress (Kim et al., 2015; Chen et al., 2021). Consistent with the preceding studies, our current study showed that the activities of SOD were decreased, while the MDA levels were increased in T2DM mice. Treatment with AS-IV reversed the decline in SOD activity, while concomitantly reducing MDA levels in the brain of T2DM mice. On the other hand, oxidative stress has also been shown to be a facilitator of neuroinflammation, which is another primary contributor to the progression of cognitive decline in T2DM (Pang et al., 2021). A clinical study showed that pro-inflammatory cytokines including TNF-α, IL-6, and IL-1β were strongly associated with poor cognitive performance in T2DM (Piatkowska-Chmiel et al., 2021) and the elevated pro-inflammatory cytokines have been reported to directly destroy the brain-blood barrier and inhibit the synaptic activity, resulting in the neuronal damage and cognitive dysfunction (Zhao et al., 2019; Michailidis et al., 2022). Moreover, AS-IV has been proven to exert potent protective effects on diabetic nephropathy *via* inhibiting the expressions of inflammatory genes in a rat model of T2DM (Gui et al., 2013a). Meanwhile, a previous study indicated that AS-IV was an effective anti-inflammatory agent in the treatment of an AD mouse model (Chen et al., 2021). In our study, the levels of TNF-α, IL-6, and IL-1β were increased in T2DM mice, while treatment with AS-IV exhibited a significantly anti-inflammatory prowess by decreasing the levels of those pro-inflammatory cytokines in T2DM mice.

Nrf2 is a transcription factor that forms a complex with Keap1 under physiological conditions. However, when oxidative stress or inflammation occurs, Nrf2 separates from Keap1, and translocates to the nucleus, inducing the expressions of antioxidant enzymes including HO-1 and NQO1, etc. (He et al., 2020). Nrf2 signaling pathway has been demonstrated to be a multifunctional signaling pathway related to anti-oxidative stress and anti-neuroinflammatory response. It has been reported that Nrf2 knockout markedly exacerbated the oxidative stress and neuroinflammatory damage and aggravated learning and memory deficits of the AD mice (Rojo et al., 2017). In addition, activation of Nrf2/HO1 signaling pathway has been demonstrated to provide neuroprotective effects against T2DM-related cognitive impairment by ameliorating oxidative stress and neuroinflammation (Zhao et al., 2019; Zhang L. et al., 2020; Pang et al., 2021). Similarly, in the present study, the expressions of Nrf2, HO-1, and NOQ1 were decreased while Keap1 was increased in T2DM mice. AS-IV administration suppress the expression of Keap1 and reversed the reduction of Nrf2, HO-1, and NOQ1. These data suggested that AS-IV ameliorated cognitive impairment in T2DM mice possibly through modulating Nrf2/Keap1/HO1/NQO1 pathway. However, we still do not know how AS-IV stimulates Nrf2, which is a limitation of this study. AS-IV may either directly or indirectly impact Nrf2, which then promotes the expression of HO-1 and NQO-1 and finally results in the improvement of neuroinflammation and oxidative stress. This will be addressed in further researches.

Overall, our study illustrated that AS-IV decreases the fasting blood glucose levels, alleviates oxidative stress and neuroinflammation, and ultimately improves cognitive impairment probably *via* Nrf2/Keap1/HO-1/NQO1 pathway in T2DM mice. It is the first time to demonstrate the neuroprotective effects of AS-IV in diabetes-related cognitive impairment, indicating that AS-IV may be a therapeutic agent for the treatment of cognitive dysfunction in T2DM.

## Data Availability Statement

The raw data supporting the conclusions of this article will be made available by the authors, without undue reservation.

## Ethics Statement

The animal study was reviewed and approved by Ethical Committee on Animal Welfare of Shanghai Jiao Tong University Affiliated Sixth People’s Hospital.

## Author Contributions

YaZ and JZ carried out the study and participated in the production of the manuscript. JF supervised the research. YaZ and YY treated the animals and preformed the animal experiment. YZhao and YuZ contributed to the discussion and analysis of the data. All authors contributed to the article and approved the submitted version.

## Funding

This study was supported by the research grants from National Natural Science Foundation of China (Grant No. 81871103, 82171179, and 81870952).

## Conflict of Interest

The authors declare that the research was conducted in the absence of any commercial or financial relationships that could be construed as a potential conflict of interest.

## Publisher’s Note

All claims expressed in this article are solely those of the authors and do not necessarily represent those of their affiliated organizations, or those of the publisher, the editors and the reviewers. Any product that may be evaluated in this article, or claim that may be made by its manufacturer, is not guaranteed or endorsed by the publisher.
